# Memory B Cells in Pregnancy Sensitization

**DOI:** 10.3389/fimmu.2021.688987

**Published:** 2021-06-30

**Authors:** Anoma Nellore, John T. Killian, Paige M. Porrett

**Affiliations:** ^1^ Department of Medicine, University of Alabama at Birmingham School of Medicine, Birmingham, AL, United States; ^2^ Department of Surgery, University of Alabama at Birmingham School of Medicine, Birmingham, AL, United States

**Keywords:** memory B cell, pregnancy, sensitization, HLA, antibody

## Abstract

Memory B cells play an important role in immunity to pathogens as these cells are poised to rapidly differentiate into antibody-secreting cells upon antigen re-encounter. Memory B cells also develop over the course of HLA-sensitization during pregnancy and transplantation. In this review, we discuss the potential contribution of memory B cells to pregnancy sensitization as well as the impact of these cells on transplant candidacy and outcomes. We start by summarizing how B cell subsets are altered in pregnancy and discuss what is known about HLA-specific B cell responses given our current understanding of fetal antigen availability in maternal secondary lymphoid tissues. We then review the molecular mechanisms governing the generation and maintenance of memory B cells during infection – including the role of T follicular helper cells - and discuss the experimental evidence for the development of these cells during pregnancy. Finally, we discuss how memory B cells impact access to transplantation and transplant outcomes for a range of transplant recipients.

## Introduction

Pregnancy represents the most common alloimmune exposure in humans. Exposure to non-self antigens of paternal origin can prime maternal B cells to generate antibodies, and maternal production of antibody against fetal antigens can occur after immunization with either minor (i.e. blood group antigens) or major antigens [i.e. human leukocyte antigen (HLA)]. Rh alloimmunization occurs when anti-D antibodies are produced in response to immunization with fetal blood (1). While this maternal anti-D antibody can cross the placenta and cause hemolytic disease of the newborn, this review will focus on the generation and consequences of antibody against the major alloantigen - HLA. As HLA alloantigens expressed by the semi-allogeneic fetus can be re-encountered on a transplanted organ from either a living or deceased donor, alloimmunization from pregnancy has particular impact for female transplant candidates and recipients. The maternal immune response to fetal alloantigens thus sets the stage for what is to come later in life and influences both access to transplantation as well as post-transplantation outcome. Despite the prevalence of pregnancy alloimmunization, the immunologic consequences of this event are very poorly understood, as pregnancy represents a unique “immunologic paradox” (2) that differs significantly from other types of immunization contexts. In this review, we discuss our current understanding of pregnancy alloimmunization with a particular focus on the generation of anti-HLA antibody and B cell memory. Herein, we use the term *pregnancy alloimmunization* to describe the response of any maternal adaptive immune subset to fetal antigen during pregnancy, whereas the term *pregnancy sensitization* refers specifically to the generation of alloantibody. In this regard, a parous woman has been alloimmunized by prior pregnancy even if she has not been sensitized (i.e. has detectable alloantibody).

## Clinical Impact of Pregnancy Sensitization

The clinical significance of pregnancy sensitization was first appreciated in the 1950s when JJ van Rood and colleagues were studying transfusion reactions in peripartum women (3). Although these investigators did not understand the structure or the etiology of the soluble factor(s) mediating cellular agglutination in their assays, this “factor” was later identified as anti-HLA antibody. This critical discovery by van Rood and colleagues impacted not only the emerging fields of transfusion medicine and organ transplantation but also allowed the development of the first reagents that were used to HLA type human tissue as well as the methodology (i.e. cytotoxicity assays). Subsequent studies which relied on cytotoxic assays later determined the prevalence and timing of pregnancy-induced anti-HLA antibody (4–6). The advent of single-antigen bead technology has greatly improved detection methods and revealed that pregnancy elicits a paternal HLA-reactive antibody response in 50-84% of mothers in the first year after pregnancy that may have HLA epitope bias (6–12). As in other types of immune exposures, the number of times that a woman is exposed to fetal alloantigen matters, as multiparous females have a higher incidence of paternal HLA-reactive antibodies (7) with strong binding to HLA epitopes (13). These anti-HLA antibodies make it more difficult for multiparous women with end organ disease to find appropriate organ donors and therefore contribute to sex-based disparities in organ transplantation (14–16). Even when transplantation in women with high levels of pre-existing anti-HLA antibody is avoided, the existence of low-level alloantibody or memory T and B cells generated by pregnancy alloimmunization may negatively impact transplant outcomes, although the specific impact of this alloimmunization event has been difficult to enumerate among other factors that influence graft outcomes (17–22). In particular, it is difficult to attribute post-transplant outcome to changes in anti-HLA antibody quantity over time as longitudinal investigation of pregnancy-induced anti-HLA antibody titers has not been performed in post-transplant recipients. Although the current available literature does not suggest that pregnancy alloimmunization promotes poor transplant outcomes *per se*, the available data sets are highly confounded. As discussion of this important topic is beyond the scope of this review, interested readers are referred to a review of pregnancy alloimmunization that discusses these data sets in detail (15). Altogether, these clinical observations underscore the importance of understanding how alloreactive B cells and antibody-secreting cells form and function during pregnancy.

## Overview of the Anatomy of Pregnancy Sensitization and the Availability of Fetal Alloantigen

To understand the mechanisms of pregnancy sensitization, it is useful to first consider the locations where maternal immune cells may encounter fetal antigen. Given our present understanding of the anatomy of pregnancy and immunity, there are several potential locations. First, immune cells in the maternal blood contact the embryo-derived trophoblast of the placenta. However, the encounter of maternal blood with placental trophoblast is not thought to significantly prime maternal B cells to produce anti-HLA antibody because human trophoblast does not express HLA-A, HLA-B, HLA-DR, HLA-DP or HLA-DQ (23–26). Nevertheless, pregnancy sensitization might still occur locally at the fetomaternal interface as maternal T and B cells encounter conceptus-derived antigens in the uterine tissue that are presented by decidual macrophages or dendritic cells. Indeed, unsupervised high dimensional flow cytometric analyses identify B cell phenotypes in uterine tissue. While immunohistochemistry demonstrates that these decidual B cells are positioned next to T cells, ectopic lymphoid follicular structures in the gravid uterus have not been formally identified. Given the importance of follicular structures in the genesis of antibody-secreting cells (discussed further below), these data suggest that anti-HLA antibody from antibody-secreting cells is unlikely to be generated in the uterus. Furthermore, *in vitro* analyses of decidual B cells *versus* peripheral B cells demonstrate an augmented ability to produce the immunoregulatory cytokine, IL-10, in the presence or absence of co-stimulatory signals (27). These data therefore suggest that the uterus may comprise a specialized tissue-resident B cell population with an as yet undetermined interaction with uterine T cells. These studies also imply IL-10 producing decidual B cell subsets arise from naïve B cell precursors locally and are maintained in an antigen-selected manner as memory after interaction with fetal trophoblast cells. In support of the latter hypothesis, *in vitro* co-culture experiments demonstrate that fetal trophoblast cells induce IL-10 production in B cells (28). Altogether, these data suggest that B cell populations in the uterus are unique and may not participate in the generation of alloantibody. This conclusion is additionally supported by emerging data suggesting that B cells play an important role in determining pregnancy outcome. For example, in murine models of pregnancy, mice that inherently lack B cells (i.e. μMT), give birth to fetuses that were smaller than wild type and with fewer regulatory T cells (29). Moreover, recurrent miscarriage has been associated with phenotypic abnormalities in the B cell compartment (30). Altogether, these data suggest that B cells in the uterus are unlikely to contribute to a large degree to the total pool of HLA antibody that is produced during pregnancy, as B cells are much more frequent in locations outside the uterus that promote the differentiation of antibody-secreting cells. We further discuss antigen availability outside the uterus below as well as our current understanding of B cell differentiation into both memory B cells and antibody-secreting cells.

**Figure 1:**
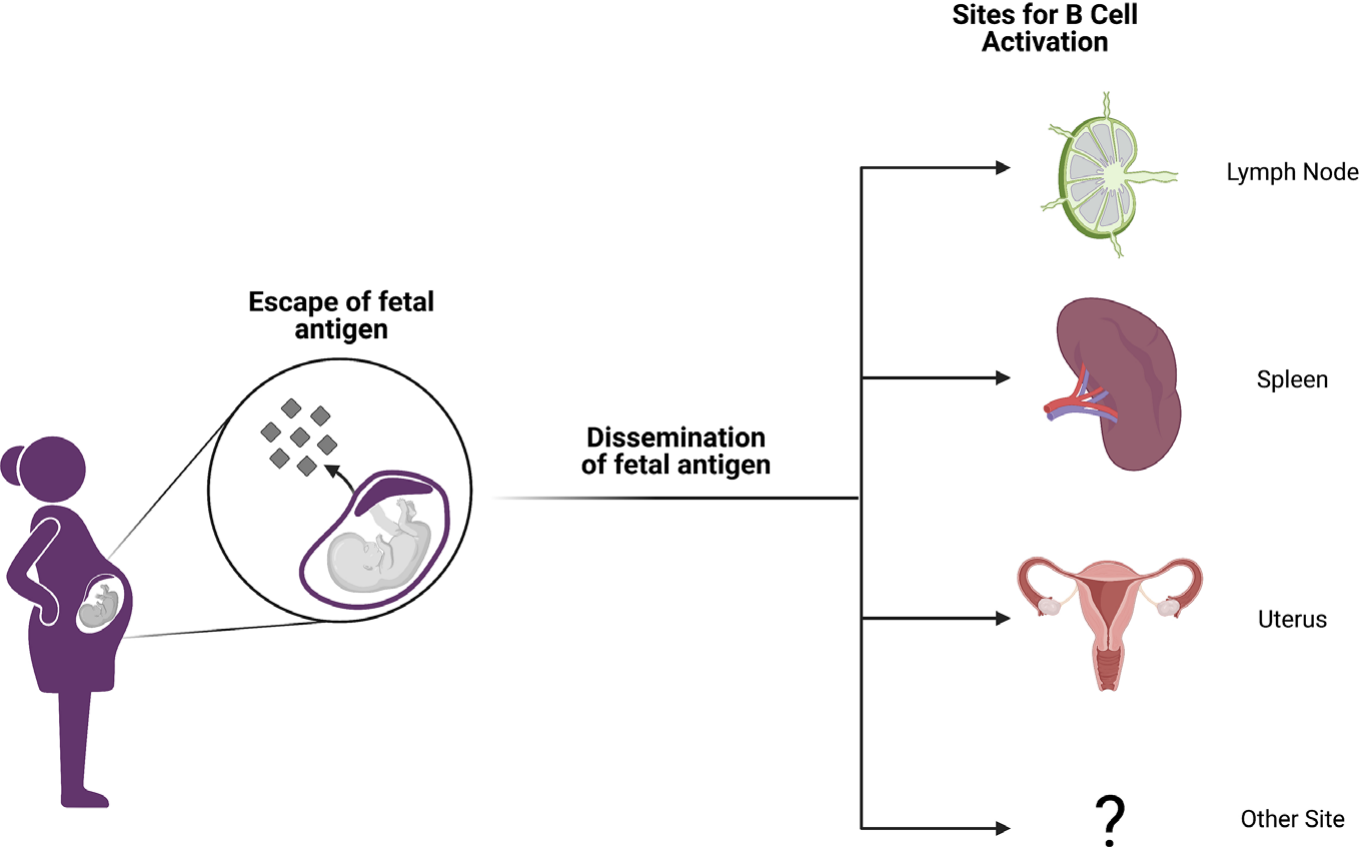
Anatomic sites of alloimmunization in pregnancy. Although fetal antigen is known to disseminate widely, it is not well understood at which sites B and T cell priming take place in allosensitization. Candidate locations include secondary lymphoid organs (SLOs) like lymph nodes and spleen. However, it is also possible that immune structures within the gravid uterus may host alloantigen encounters or perhaps other anatomic sites are involved. Figure created with BioRender.com.

In light of the findings discussed above, it is likely that the majority of pregnancy alloimmunization occurs at sites distant from the fetomaternal interface (**Figure 1**). It is indeed now well established in both mice and humans that conceptus-derived cells, proteins, exosomes, RNA, and DNA disseminate broadly in the maternal circulation and can deposit in maternal tissues (31–36). Importantly, disseminated protein antigens are detectable in the maternal spleen and other secondary lymphoid organs in mouse models of pregnancy (37) where these antigens can prime both maternal T cells (37–45) and B cells (46). Although fetal microchimerism clearly occurs, resulting in seeding of maternal tissues that may persist for decades, its impact on humoral immunity remains undefined (32, 36). In mothers, the development of regulatory T cells may be influenced by the presence of fetal microchimerism, and this T cell literature is excellently reviewed by Kinder et al. (47) However, the persistence of circulating anti-HLA antibodies with specificity for fetal HLA alleles and their vigorous recall response following transplant suggest that the B cell arm of the adaptive response is not similarly skewed towards a regulatory phenotype (48).

Although it is possible that antigen presenting cells from the uterus may traffic to the uterine draining lymph node and prime maternal T and B cells as well, mouse studies suggest that such egress of maternal antigen presenting cells from the uterus is impaired during pregnancy (49). Collectively, these data suggest that pregnancy alloimmunization primarily occurs in secondary lymphoid organs such as the spleen and lymph nodes, perhaps after uptake of circulating antigen by antigen presenting cells within these sites. However, many knowledge gaps remain. It is important to note that the majority of these data have yet to be validated in humans owing to the extreme polymorphism of HLA and the lack of HLA-specific reagents. Moreover, it is unclear how effective B cell help is provided by maternal T cells given that pregnancy alloimmunization often propels the expansion of hypofunctional and/or suppressive regulatory T cell populations.

## Generation of Memory B Cells and Antibodies

To understand B cells responses in pregnancy, it is first useful to review what is known about B cell activation and differentiation in the context of infection and immunization, where B cell biology has been better studied. In this section, we review the foundation of humoral responses by outlining the pathways of B cell activation and the subsequent production of memory B cells and antibody-secreting cells.

### B Cell Activation Pathways

Naïve B cells circulate through the secondary lymphoid organs in search of their cognate antigen in either soluble or membrane-bound form (50). While naïve B cells may be activated outside of secondary lymphoid organs in ectopic lymphoid structures (51) or in mice lacking organized lymph nodes or spleens (52), secondary lymphoid organs represent the chief location for initial antigen encounter and B cell activation (53). Although B cell receptors are able to recognize intact, soluble antigen, multiple mechanisms exist to concentrate and present antigen for B cells. Afferent lymph enters a lymph node at the subcapsular sinus, where subcapsular sinus macrophages capture and present complement-opsonized antigen to B cells (54). Non-opsonized antigen may reach the medulla of the lymph node where it is bound by medullary macrophages or dendritic cells, phagocytosed, and presented or transported to the follicle (55). Within the follicle, follicular dendritic cells are specialized for the task of presenting antigen to B cells, capturing opsonized antigen and recycling it in a non-degradative endosomal compartment that facilitates long-term antigen presentation within a secondary follicle’s germinal center (GC) response (56). How antigen is captured and presented in the context of pregnancy is unknown.

**Figure 2:**
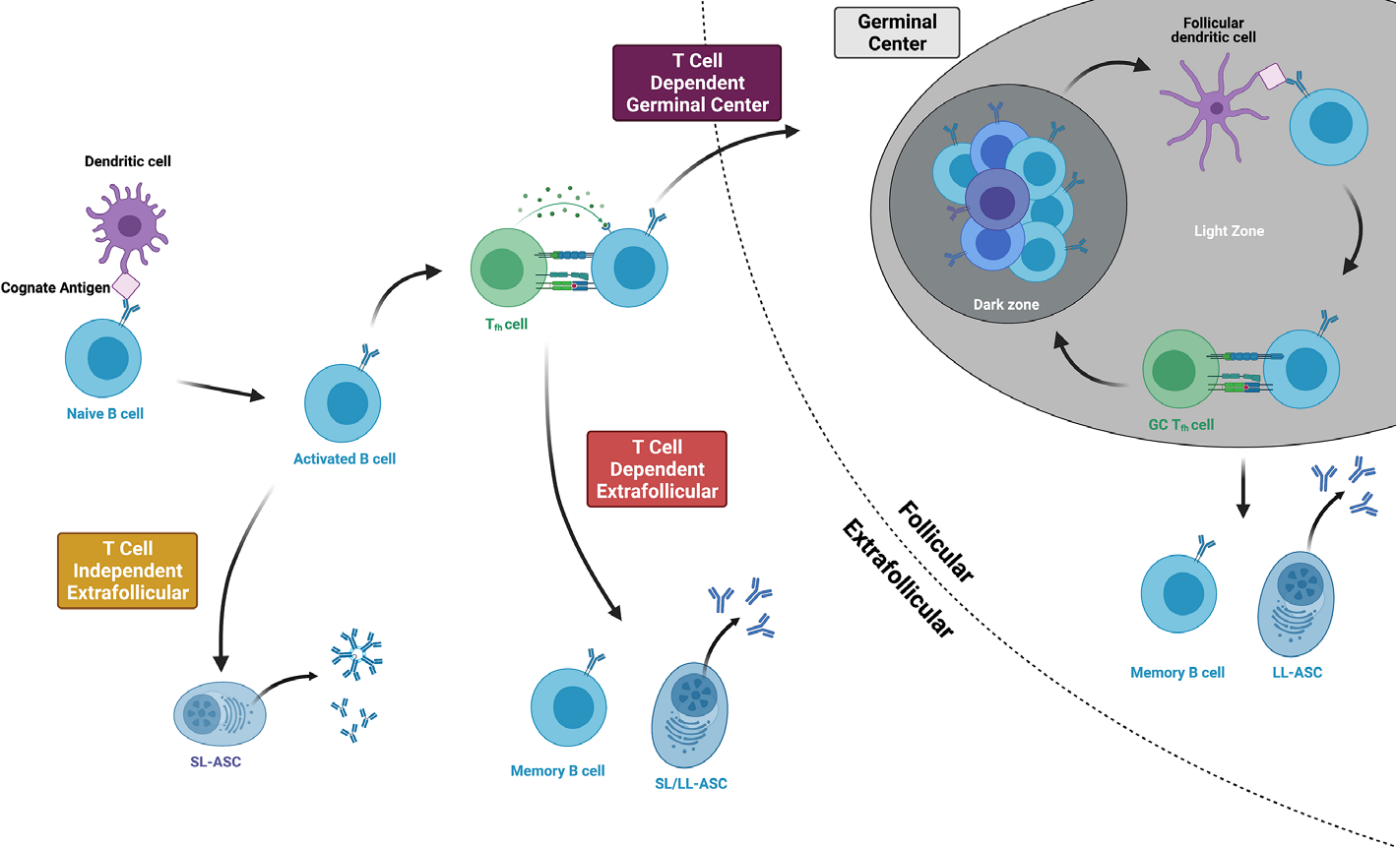
Three main pathways of B cell activation and differentiation. Protein antigens promote T cell dependent responses, culminating in either germinal center or extrafollicular pathways that produce long-lived memory B cells and antibody-secreting cells (ASCs). Within the germinal center, B cells cycle between the dark and light zones, proliferating, mutating, and undergoing affinity maturation. T cell independent antigens include mitogens and highly repetitive polysaccharides that can stimulate B cell responses without T cell help. Figure created with BioRender.com.

Following the naïve B cell’s initial encounter with its cognate antigen, the B cell becomes activated and follows one of three major pathways, as indicated by the i, ii, iii numeric identifiers below (**Figure 2**). The B cell follows chemokine and oxysterol gradients to migrate to the interface of the B cell follicle and the T cell zone (T:B border), upregulating the receptors Epstein-Barr virus-induced molecule 2 (EBI2) (57) and C-C chemokine receptor 7 (CCR7) while maintaining C-X-C motif chemokine receptor 5 (CXCR5) expression (58). Some B cells then enter the germinal center (GC) while others remain in the extrafollicular (EF) region. In the germinal center (also known as the secondary follicle), somatic hypermutation and clonal selection produce high-affinity memory B cells and long-lived antibody-secreting cells in a T-dependent process (i). In the extrafollicular region, naïve B cells differentiate into memory B cells or antibody-secreting cells *via* T cell-dependent (ii) or T-independent processes (iii) (59). Although the spectrum of antigen that might prime B cell responses in pregnancy is unknown, fragments of HLA proteins are likely to be involved in the priming of anti-HLA antibody. We thus will focus on the first two pathways (i, ii) given that B cell priming by protein antigens is a T-dependent process (60, 61), and anti-HLA antibody is known to persist for decades in some women after pregnancy sensitization, thus implying the differentiation of long-lived antibody-secreting cells.

At the T:B border, B cells present their antigen and receive CD4^+^ T cell help from developing T follicular helper cells (Tfhs). Tfhs, whose primary purpose is to help activated B cells become memory B cells or long-lived antibody-secreting cells, differentiate from naïve CD4^+^ T cells after being primed by dendritic cells in the lymph node or spleen (62). They express the Tfh lineage-defining transcription factor *Bcl6* and the chemokine receptor CXCR5 and then migrate to the T:B border to interact with activated B cells (63). Here, B cells provide the key second step in Tfh differentiation by expressing ICOS ligand (ICOSL) and presenting the Tfh’s cognate antigen *via* major histocompatibility complex class II (MHC class II) proteins (64). At the same time, the Tfh provides the B cell with critical support. Tfh expression of CD40 ligand (CD40L) delivers the second signal of B cell activation following ligation of the B cell receptor (65), and cytokines like IL-21 and IL-4 facilitate both T-dependent extrafollicular and germinal cell responses (66). From the T:B border, Tfhs then enter the germinal center, becoming germinal center Tfhs (GC-Tfhs) (67). Of note, others use the nomenclature *pre-Tfh* or *extrafollicular mantle Tfh* to refer to BCL-6^+^ CD4^+^ T cells outside of the germinal center and simply *Tfh* for those within a germinal center structure (67, 68).

Following these events at the T:B border, B cells briefly proliferate in the outer follicle before making a fate decision to enter the germinal center or follow an extrafollicular pathway (69). The affinity of the B cell receptor, cytokine milieu, Tfh interactions, and innate sensors all contribute to this decision (70). High affinity B cell clones experiencing B cell receptor crosslinking may favor an extrafollicular response (71). Although B cells must meet a relative affinity threshold in order to effectively capture antigen and engage Tfhs at the T:B border (72), this threshold may be sufficiently low that it does not provide a considerable barrier to germinal center entrance among naïve antigen-specific clones (73). Tfhs assist activated B cells by indirectly assessing affinity and secreting IL-21 (66). and pathogen- or damage-associated patterns that signal through toll-like receptor 9 (TLR9) promote rapid antibody production by inducing the differentiation of antibody-secreting cells while decreasing B cell antigen presentation and thus, the effectiveness of B cell and T cell cooperation (74). Overall, these fate decisions correlate with EBI2 expression, whose loss guides the activated B cell into the germinal center and whose persistence corresponds with an extrafollicular response (75).

### Germinal Center Responses

Activated B cells that enter the germinal center participate in the immunological equivalent of natural selection, resulting in a population of memory B cells and long-lived antibody-secreting cells that maintain long-lasting memory. Germinal centers feature two histologic compartments: a light zone where follicular dendritic cells present antigen for B cells (76) and Tfhs positively select higher-affinity B cell clones and a dark zone where B cells proliferate and acquire immunoglobulin gene somatic mutations (77). In the light zone, cognate B cells and Tfhs become “entangled” *via* multiple cell surface receptor interactions (78) with Tfhs providing B cell help *via* CD40L signaling and cytokines including IL-4, IL-21, and BAFF (79). B cells then migrate to the dark zone, where expression of the enzyme Activation-induced Cytidine Deaminase (AID) generates uracil bases which may be replaced with thymine bases leading to somatic hypermutation (80). These newly mutated germinal center B cells proliferate and return to the light zone to compete for antigen from follicular dendritic cells and re-engage Tfhs, with higher affinity B cells receiving continued Tfh help (79). It should be noted that several traditionally-held views about germinal centers have been challenged by new research. One such view, which holds that class-switch recombination occurs within the germinal center has been challenged by data showing that the majority of naïve B cells undergo class-switch recombination prior to germinal center entry and that class-switch recombination may in fact be repressed within the germinal center (81). Another view, which holds that somatic hypermutation and memory develop solely within the germinal center is addressed in the following paragraph, noting the existence of these processes in extrafollicular responses. Work continues to elucidate the factors that control the fate decision of a germinal center B cell to become a memory B cell or a long-lived antibody-secreting cell. Lower affinity B cell receptors may favor memory B cell development, as B cells receiving less Tfh help maintain high expression of the transcriptional repressor *Bach2* and subsequently become memory B cells (82). In addition to affinity, timing may also play a role in fate decisions, with the early germinal center response predominantly yielding memory B cells and the late germinal center response producing long-lived antibody-secreting cells (83). Overall, these fate decisions are consistent with observations that memory B cells may overall be more broadly reactive and of lower affinity than long-lived antibody-secreting cells (84, 85).

### Extrafollicular Responses

**Figure 3:**
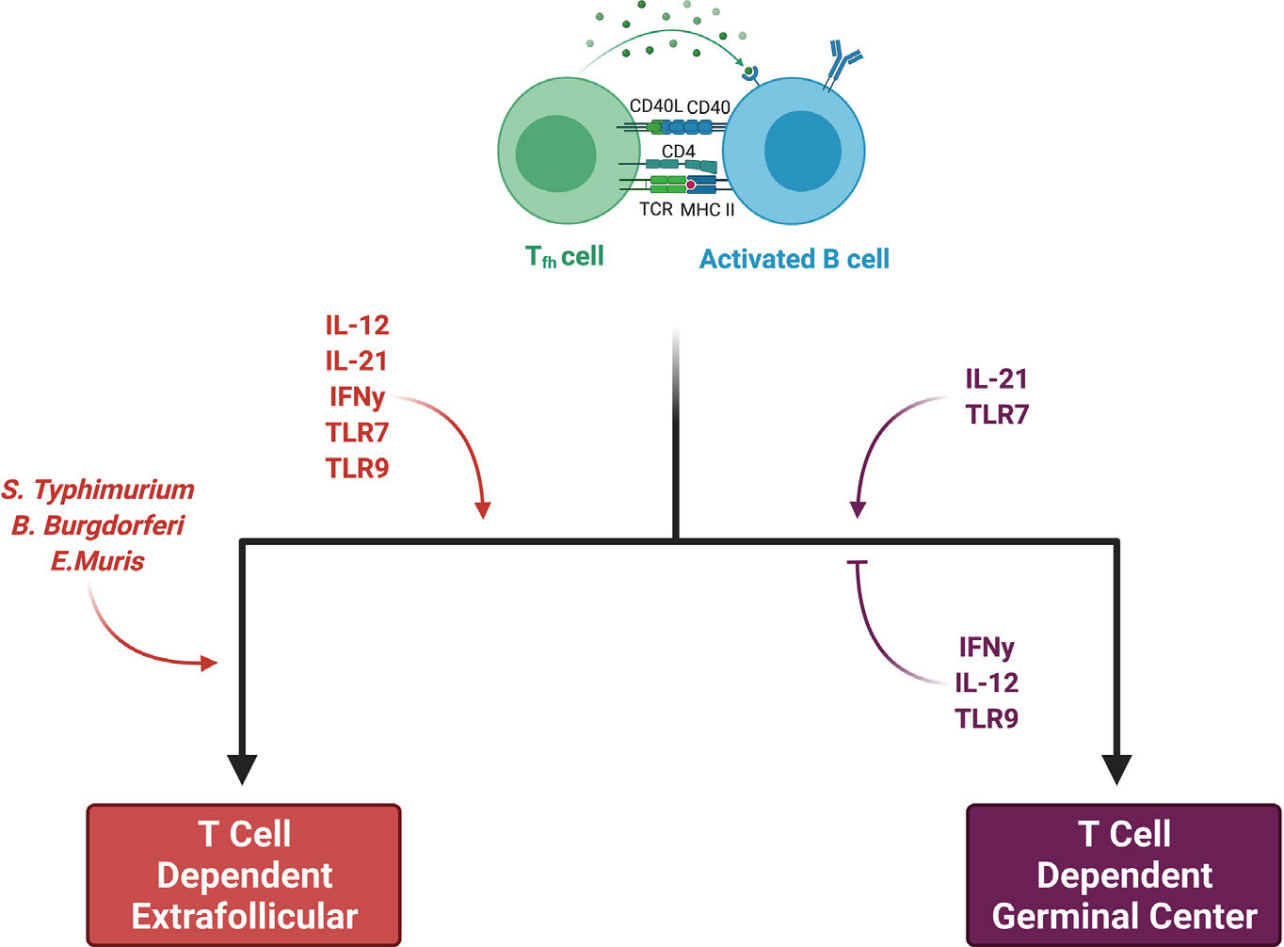
Factors skewing responses toward either germinal center or extrafollicular pathways. Both pathogen and host factors contribute to a B cell’s decision between an extrafollicular (EF) and germinal center (GC) response. Certain bacteria promote an EF response through collapse of secondary lymphoid organ (SLO) architecture. While IL-21 and TLR7 may promote both EF and GC responses, IL-12, IFN-y, and TLR9 all favor EF responses. IL-12 suppresses Tfh development while favoring short-lived ASC production. INF-y may have the same effects on Tfh suppression. TLR9 clearly stimulates EF responses, whereas it may inhibit GC responses, at least in autoimmune diseases. Figure created with BioRender.com.

Some activated B cells do not enter the germinal center reaction and instead proliferate and differentiate *via* an extrafollicular pathway. Recent work utilizing a semiallogeneic mouse model of pregnancy suggests that the maternal B cell response to fetal antigens may proceed in a fashion independent of the germinal center, which may implicate the extrafollicular pathway in the development of maternal sensitization (46). In an excellent review, Elsner et al. distinguish between canonical B cell responses, in which a brief extrafollicular phase precedes the germinal center response, and non-canonical ones, which have extended extrafollicular phases (86). Although the factors which skew a response towards the non-canonical extrafollicular pathway are an area of active investigation, it appears that both pathogen and host factors play important roles (**Figure 3**). *Salmonella Typhimurium, Borrelia Burgdorferi, and Ehrlichia Muris* promote the development of a non-canonical extrafollicular response *via* lipopolysaccharide (LPS)- and tumor necrosis factor-α (TNF-α)-mediated collapse of traditional lymph node and splenic architecture (87, 88). Innate sensors and cytokines play a role in extrafollicular responses as well, as studies of systemic lupus erythematosus have shown that B cell TLR7 hyperresponsiveness and IL-21 signaling synergize to generate autoreactive antibody-secreting cells outside of germinal centers (89). Although it appears that only germinal centers can generate IgG-secreting long-lived antibody-secreting cells (90, 91), mice lacking germinal centers are still capable of generating IgM-secreting long-lived antibody-secreting cells that home to the spleen as opposed to the bone marrow (92). Aside from these differences in the output of antibody-secreting cells, extrafollicular responses are still capable of inducing AID expression, yielding somatic hypermutation and class-switch recombination, and generating antigen-specific memory B cells (87, 93, 94). Consequently, it will be important in future work studying pregnancy sensitization in both humans and mice to determine the isotype of the fetal-specific or anti-HLA antibodies which are detected. This information will allow us to draw inferences about whether B cell responses originate within the follicle (i.e. germinal center) or outside the follicle (i.e. extrafollicular response). Discrimination between these two pathways has significant implications for multiple facets of pregnancy sensitization, including the durability of the antibody that is generated.

## Function of Memory B Cell and Antibody-Secreting Cell Responses

Memory B cells provide the host protection from subsequent pathogen challenge through mechanisms that largely complement long-lived antibody-secreting cells. Importantly, multiple studies have shown that the reactivities of memory B cells and antibody-secreting cells do not overlap perfectly (85, 95). For example, memory B cells produced in response to certain viral infections are more broadly reactive than the antibodies produced by antibody-secreting cells, and these memory B cells may cross-react with different epitopes on other viral strains, affording protection from variants that are not neutralized by the antibodies produced by antibody-secreting cells (84, 85, 96). Memory B cell recall responses are facilitated by B cell receptor reactivity that is broader than the reactivity of long-lived antibody-secreting cells but is still antigen-specific, and memory B cells can rapidly respond to a second infection by differentiating into antibody-secreting cells or by entering germinal centers where they may affinity-mature, switch isotypes, and emerge as new memory B cells or high-affinity antibody-secreting cells (97). Although a mouse model with homologous boosting showed that memory B cells infrequently reentered germinal centers (98), humans respond to heterologous boosting with broadly reactive memory B cells that rapidly differentiate into antibody-secreting cells and reenter germinal centers (99). In addition to B cell receptor affinity, multiple other factors regulate the memory B cell recall response. Circulating antibodies (generated by long-lived antibody-secreting cells) may bind epitopes that memory B cells would otherwise recognize, effectively inhibiting a memory B cell recall response against those bound, cognate epitopes while promoting a response against exposed epitopes (100). The isotype of the memory B cells also appears to indicate its fate preference during a recall response, as both mouse and human IgM+ memory B cells show a propensity to reenter germinal centers, whereas IgG+ memory B cells are more likely to differentiate into antibody-secreting cells (101, 102). It is not clear whether the isotype of the B cell receptor mediates this effect through properties related to intracellular domains of the B cell receptor or as a result of cell-intrinsic properties generated at the time of memory B cell formation.

## HLA-Reactive B Cells

HLA-reactive B cells have been studied following both pregnancy and transplant, revealing insights into their frequency, persistence, and specificity. Three techniques have been used in humans to study HLA-reactive B cells: 1) ELISPOT, 2) polyclonal activation, and 3) fluorochrome-labeled HLA tetramers. Following pregnancy or transplant, HLA-reactive memory B cells are detectable by ELISPOT at frequencies of approximately 1 in 1,000 to 1 in 40,000 B cells, and these cells are notably absent from unsensitized individuals (103–106). Studies that polyclonally activated circulating HLA-reactive memory B cells and studied their supernatants have shown a restricted number of specificities as compared to circulating anti-HLA antibodies. However, “hidden sensitization,” which is the presence of memory B cells which target HLA specificities that are not represented among circulating anti-HLA antibodies, is estimated to exist in perhaps 40% of sensitized patients (107, 108). Like ELISPOT, HLA tetramers have been used to estimate frequencies of HLA-reactive B cells following a sensitizing event. However, in light of data showing that up to 6% of B cells in sensitized humans and 1% of B cells in unsensitized humans bind such tetramers, the assay may lack specificity (109, 110). Notably, other investigators who have studied women sensitized through pregnancy have shown significantly lower frequencies, estimating that roughly 1 in 10,000 memory B cells is HLA-reactive (13). Concerns about the specificity of tetramer-binding B cells are likely justified, given that fewer than 30% of antibodies expressed from these tetramer-positive B cells bind the HLA molecule of interest, a finding consistent for both HLA class I and class II tetramers (13, 111). While these data therefore showcase how HLA-reactive memory B cells in humans can be captured and characterized, it is clear that available assays provide a limited view of prevalence and specificity. Given that the detection of HLA-reactive B cells in transplant patients carries prognostic significance (112), it is critical that we better understand the biology of these cells and close the significant gaps that remain in our understanding of how and where these cells differentiate, the signals governing their differentiation, and the molecular and epigenetic programs that guide this differentiation in humans. In summary, we conclude that although HLA-reactive B cells can be detected using current assays, their prevalence and the breadth of their anti-HLA repertoire may be underestimated given the use of small volume peripheral blood samples. Future work that pairs the above techniques with more advanced sampling, phenotyping, and antibody cloning approaches from vaccine (99, 113) and infectious disease studies (114) will enhance our understanding of HLA-reactive memory B cells and may provide insights that allow for better diagnosis and treatment of anti-HLA responses in transplant.

Murine models of pregnancy and allotransplantation have been used to overcome some of these limitations, and mouse investigations have thus revealed several insights into the origins and functions of alloreactive B cells (46, 115). Pertinent to this review, a recent study used peptide:MHC tetramers to examine alloreactive T and B cells concordantly in mice alloimmunized by either pregnancy or transplantation. The authors mated congenic mice and then performed allogeneic heart transplantation with and without co-stimulation blockade on the postpartum female partner to assess the role of pregnancy-induced alloimmunity on transplant outcome. The authors found that postpartum mice developed fetal alloreactive B and T cell responses and that the alloreactive T cell response remained tolerogenic after secondary heart allotransplantation. Next, the authors performed experiments on animals that lacked circulating immunoglobulins (sIgKO) or animals that lacked immunoglobulins and B cells (μMT) to dissect the relative roles of pregnancy-induced alloreactive memory B cells *versus* antibody-secreting cells in precipitating allograft rejection. Postpartum μMT animals were able to spontaneously accept allogenic heart transplants long-term without any immunosuppressive medication, suggesting that the alloreactive humoral immune arm that develops after pregnancy is critical to mediating subsequent organ transplant rejection. This spontaneous acceptance was lost when postpartum sIgKO were given allogeneic heart transplants, suggesting that even in the absence of circulating fetal specific antibody, a subset of alloreactive memory B cells elicited by pregnancy and recalled after transplant, was sufficient to abrogate tolerance. What subset of memory B cells mediates this break in tolerance and the mechanisms by which this occurs remain to be elucidated.

## Lessons From Memory B Cells in Other Immune States

Although the authors do not perform molecular characterization of the alloreactive memory B cell compartment that develops in these postpartum mice and is recalled after subsequent allotransplantation, the authors perform limited immunophenotyping. Despite the presence of circulating fetal-specific antibody, the alloreactive B cells of postpartum animals were found to lack classic germinal center markers in uterine-draining lymph nodes. Only after subsequent heart allotransplantation did the alloreactive B cells of postpartum animals express classical germinal center markers. Thus, the alloreactive B cells in postpartum animals after primary pregnancy appear to arise in extrafollicular reactions and have memory properties as they can be recalled after allotransplantation, present antigen to donor-specific T cells and form secondary antibody-secreting cells in germinal center reactions. These data are interesting in the context of recent insights into the functional diversity of antigen-specific memory B cells from the autoimmune (89) and immunization literature (116, 117) which have identified certain transcriptionally distinct antigen-specific memory B cells which are poised to directly form antibody-secreting cells in extrafollicular reactions (effectors) *versus* others that are recalled into germinal center reactions in lymphoid tissues to undergo affinity maturation and produce daughter antibody-secreting cells (effector memory) and daughter memory B cells (central memory). It will be important for future studies of the pregnancy-induced alloreactive immune response to clarify the transcriptional and functional diversity of this response in order to understand if particular alloreactive memory B cell subsets that develop after pregnancy sensitization are more or less important to mediating subsequent allograft rejection.

## Conclusions

While our knowledge of B cell responses in pregnancy is underdeveloped, recent work suggests that fundamental mechanisms underlying B cell responses in infection are generalizable to the pregnancy setting. The further development of reagents that can track antigen-specific B cell responses in humans will be critical to improve our understanding of B cell differentiation and fate after pregnancy alloimmunization, including the generation of memory B cells, as well as short-lived and long-lived antibody-secreting cells. An improved understanding of these cell types and the mechanisms by which they arise will be critical to improve transplant access and outcomes among female transplant recipients and alleviate the significant sex disparity that exists in organ transplantation.

## Author Contributions

All authors contributed to the article and approved the submitted version.

## Conflict of Interest

The authors declare that the research was conducted in the absence of any commercial or financial relationships that could be construed as a potential conflict of interest.

## References

[1] Webb J Delaney M. Red Blood Cell Alloimmunization in the Pregnant Patient. Transfus Med Rev (2018) 32(4):213–9. 10.1016/j.tmrv.2018.07.002 30097223

[2] Medawar PB. Some Immunological and Endocrinological Problems Raised by the Evolution of Viviparity in Vertebrates. Symp Soc Exp Biol (1953) 7:320–38.

[3] Van Rood JJ Eernisse JG Van Leeuwen A. Leucocyte Antibodies in Sera From Pregnant Women. Nature (1958) 181(4625):1735–6. 10.1038/1811735a0 13566127

[4] Regan L Braude PR Hill DP. A Prospective Study of the Incidence, Time of Appearance and Significance of Anti-Paternal Lymphocytotoxic Antibodies in Human Pregnancy. Hum Reprod (1991) 6(2):294–8. 10.1093/oxfordjournals.humrep.a137325 2056027

[5] Triulzi DJ Kleinman S Kakaiya RM Busch MP Norris PJ Steele WR. The Effect of Previous Pregnancy and Transfusion on HLA Alloimmunization in Blood Donors: Implications for a Transfusion-Related Acute Lung Injury Risk Reduction Strategy. Transfusion (2009) 49(9):1825–35. 10.1111/j.1537-2995.2009.02206.x PMC2841001 19453983

[6] van Kampen CA Versteeg-vd Voort Maarschalk MF Langerak-Langerak J Roelen DL Claas FH. Kinetics of the Pregnancy-Induced Humoral and Cellular Immune Response Against the Paternal HLA Class I Antigens of the Child. Hum Immunol (2002) 63(6):452–8. 10.1016/s0198-8859(02)00396-8 12039520

[7] Honger G Fornaro I Granado C Tiercy JM Hosli I Schaub S. Frequency and Determinants of Pregnancy-Induced Child-Specific Sensitization. Am J Transplant (2013) 13(3):746–53. 10.1111/ajt.12048 23311303

[8] Honger G Niemann M Schawalder L Jones J van Heck MR van de Pasch LAL. Toward Defining the Immunogenicity of HLA Epitopes: Impact of HLA Class I Eplets on Antibody Formation During Pregnancy. HLA (2020) 96(5):589–600. 10.1111/tan.14054 32829523

[9] Masson E Vidal C Deschamps M Bongain S Thevenin C Dupont I. Incidence and Risk Factors of Anti-HLA Immunization After Pregnancy. Hum Immunol (2013) 74(8):946–51. 10.1016/j.humimm.2013.04.025 23628391

[10] Lee J Romero R Xu Y Miranda J Yoo W Chaemsaithong P. Detection of Anti-HLA Antibodies in Maternal Blood in the Second Trimester to Identify Patients at Risk of Antibody-Mediated Maternal Anti-Fetal Rejection and Spontaneous Preterm Delivery. Am J Reprod Immunol (2013) 70(2):162–75. 10.1111/aji.12141 PMC4154511 23841577

[11] Kussel L Herkner H Wahrmann M Eskandary F Doberer K Binder J. Longitudinal Assessment of HLA and MIC-a Antibodies in Uneventful Pregnancies and Pregnancies Complicated by Preeclampsia or Gestational Diabetes. Sci Rep (2017) 7(1):13524. 10.1038/s41598-017-13275-6 29051520 PMC5648869

[12] Vilches M Nieto A. Analysis of Pregnancy-Induced Anti-Hla Antibodies Using Luminex Platform. Transplant Proc (2015) 47(9):2608–10. 10.1016/j.transproceed.2015.09.032 26680049

[13] Kramer CSM Franke-van Dijk MEI Bakker KH Uyar-Mercankaya M Karahan GE Roelen DL. Generation and Reactivity Analysis of Human Recombinant Monoclonal Antibodies Directed Against Epitopes on HLA-DR. Am J Transplant (2020) 20(12):3341–53. 10.1111/ajt.15950 PMC7754395 32342632

[14] Bromberger B Spragan D Hashmi S Morrison A Thomasson A Nazarian S. Pregnancy-Induced Sensitization Promotes Sex Disparity in Living Donor Kidney Transplantation. J Am Soc Nephrol (2017) 28(10):3025–33. 10.1681/ASN.2016101059 PMC5619956 28483798

[15] Porrett PM. Biologic Mechanisms and Clinical Consequences of Pregnancy Alloimmunization. Am J Transplant (2018) 18(5):1059–67. 10.1111/ajt.14673 29369525

[16] Redfield RR Scalea JR Zens TJ Mandelbrot DA Leverson G Kaufman DB. The Mode of Sensitization and its Influence on Allograft Outcomes in Highly Sensitized Kidney Transplant Recipients. Nephrol Dial Transplant (2016) 31(10):1746–53. 10.1093/ndt/gfw099 27387475

[17] Pollack MS Trimarchi HM Riley DJ Casperson PR Manyari LE Suki WN. Shared Cadaver Donor-Husband HLA Class I Mismatches as a Risk Factor for Renal Graft Rejection in Previously Pregnant Women. Hum Immunol (1999) 60(11):1150–5. 10.1016/s0198-8859(99)00104-4 10600014

[18] Ghafari A. Offspring-to-Mother and Husband-to-Wife Renal Transplantation: A Single-Center Experience. Transplant Proc (2008) 40(1):140–2. 10.1016/j.transproceed.2007.11.062 18261570

[19] Mahanty HD Cherikh WS Chang GJ Baxter-Lowe LA Roberts JP. Influence of Pretransplant Pregnancy on Survival of Renal Allografts From Living Donors. Transplantation (2001) 72(2):228–32. 10.1097/00007890-200107270-00010 11477343

[20] Miles CD Schaubel DE Liu D Port FK Rao PS. The Role of Donor-Recipient Relationship in Long-Term Outcomes of Living Donor Renal Transplantation. Transplantation (2008) 85(10):1483–8. 10.1097/TP.0b013e3181705a0f 18497690

[21] Choi JY Kwon OJ Kang CM. The Effect of Donor-Recipient Relationship on Long-Term Outcomes of Living Related Donor Renal Transplantation. Transplant Proc (2012) 44(1):257–60. 10.1016/j.transproceed.2011.11.017 22310626

[22] Cohen JB Owei L Sawinski DL Porrett PM. Inferior Long-Term Allograft and Patient Outcomes Among Recipients of Offspring Living Donor Kidneys. Am J Transplant (2018) 18(7):1699–709. 10.1111/ajt.14631 PMC6013327 29266831

[23] Hunt JS Fishback JL Chumbley G Loke YW. Identification of Class I Mhc Mrna in Human First Trimester Trophoblast Cells by *in Situ* Hybridization. J Immunol (1990) 144(11):4420–5. 2341725

[24] Peyman JA Hammond GL. Localization of IFN-Gamma Receptor in First Trimester Placenta to Trophoblasts But Lack of Stimulation of HLA-DRA, -DRB, or Invariant Chain Mrna Expression by IFN-Gamma. J Immunol (1992) 149(8):2675–80. 1401907

[25] King A Burrows TD Hiby SE Bowen JM Joseph S Verma S. Surface Expression of HLA-C Antigen by Human Extravillous Trophoblast. Placenta (2000) 21(4):376–87. 10.1053/plac.1999.0496 10833373

[26] Apps R Murphy SP Fernando R Gardner L Ahad T Moffett A. Human Leucocyte Antigen (HLA) Expression of Primary Trophoblast Cells and Placental Cell Lines, Determined Using Single Antigen Beads to Characterize Allotype Specificities of Anti-HLA Antibodies. Immunology (2009) 127(1):26–39. 10.1111/j.1365-2567.2008.03019.x 19368562 PMC2678179

[27] Benner M Feyaerts D Garcia CC Inci N Lopez SC Fasse E. Clusters of Tolerogenic B Cells Feature in the Dynamic Immunological Landscape of the Pregnant Uterus. Cell Rep (2020) 32(13):108204. 10.1016/j.celrep.2020.108204 32997982

[28] Guzman-Genuino RM Dimova T You Y Aldo P Hayball JD Mor G. Trophoblasts Promote Induction of a Regulatory Phenotype in B Cells That can Protect Against Detrimental T Cell-Mediated Inflammation. Am J Reprod Immunol (2019) 82(6):e13187. 10.1111/aji.13187 31487409 PMC8232043

[29] Busse M Campe KJ Nowak D Schumacher A Plenagl S Langwisch S. Il-10 Producing B Cells Rescue Mouse Fetuses From Inflammation-Driven Fetal Death and are Able to Modulate T Cell Immune Responses. Sci Rep (2019) 9(1):9335. 10.1038/s41598-019-45860-2 31249364 PMC6597542

[30] Carbone J Sarmiento E Gallego A Lanio N Navarro J Garcia S. Peripheral Blood T- and B-Cell Immunophenotypic Abnormalities in Selected Women With Unexplained Recurrent Miscarriage. J Reprod Immunol (2016) 113:50–3. 10.1016/j.jri.2015.11.003 26686770

[31] Bianchi DW Mahr A Zickwolf GK Houseal TW Flint AF Klinger KW. Detection of Fetal Cells With 47,XY,+21 Karyotype in Maternal Peripheral Blood. Hum Genet (1992) 90(4):368–70. 10.1007/BF00220460 1483692

[32] Bianchi DW Zickwolf GK Weil GJ Sylvester S DeMaria MA. Male Fetal Progenitor Cells Persist in Maternal Blood for as Long as 27 Years Postpartum. Proc Natl Acad Sci USA (1996) 93(2):705–8. 10.1073/pnas.93.2.705 PMC40117 8570620

[33] Lo YM Corbetta N Chamberlain PF Rai V Sargent IL Redman CW. Presence of Fetal DNA in Maternal Plasma and Serum. Lancet (1997) 350(9076):485–7. 10.1016/S0140-6736(97)02174-0 9274585

[34] Tay CS Tagliani E Collins MK Erlebacher A. Cis-Acting Pathways Selectively Enforce the non-Immunogenicity of Shed Placental Antigen for Maternal CD8 T Cells. PloS One (2013) 8(12):e84064. 10.1371/journal.pone.0084064 24391885 PMC3877187

[35] Mitchell MD Peiris HN Kobayashi M Koh YQ Duncombe G Illanes SE. Placental Exosomes in Normal and Complicated Pregnancy. Am J Obstet Gynecol (2015) 213(4 Suppl):S173–81. 10.1016/j.ajog.2015.07.001 26428497

[36] Rijnink EC Penning ME Wolterbeek R Wilhelmus S Zandbergen M van Duinen SG. Tissue Microchimerism is Increased During Pregnancy: A Human Autopsy Study. Mol Hum Reprod (2015) 21(11):857–64. 10.1093/molehr/gav047 26307194

[37] Erlebacher A Vencato D Price KA Zhang D Glimcher LH. Constraints in Antigen Presentation Severely Restrict T Cell Recognition of the Allogeneic Fetus. J Clin Invest (2007) 117(5):1399–411. 10.1172/JCI28214 PMC1849983 17446933

[38] Tafuri A Alferink J Moller P Hammerling GJ Arnold B. T Cell Awareness of Paternal Alloantigens During Pregnancy. Science (1995) 270(5236):630–3. 10.1126/science.270.5236.630 7570020

[39] Jiang SP Vacchio MS. Multiple Mechanisms of Peripheral T Cell Tolerance to the Fetal “Allograft”. J Immunol (1998) 160(7):3086–90. 9531261

[40] Rowe JH Ertelt JM Aguilera MN Farrar MA Way SS. Foxp3(+) Regulatory T Cell Expansion Required for Sustaining Pregnancy Compromises Host Defense Against Prenatal Bacterial Pathogens. Cell Host Microbe (2011) 10(1):54–64. 10.1016/j.chom.2011.06.005 21767812 PMC3140139

[41] Rowe JH Ertelt JM Xin L Way SS. Pregnancy Imprints Regulatory Memory That Sustains Anergy to Fetal Antigen. Nature (2012) 490(7418):102–6. 10.1038/nature11462 PMC3465465 23023128

[42] Samstein RM Josefowicz SZ Arvey A Treuting PM Rudensky AY. Extrathymic Generation of Regulatory T Cells in Placental Mammals Mitigates Maternal-Fetal Conflict. Cell (2012) 150(1):29–38. 10.1016/j.cell.2012.05.031 22770213 PMC3422629

[43] Kalekar LA Schmiel SE Nandiwada SL Lam WY Barsness LO Zhang N. Cd4(+) T Cell Anergy Prevents Autoimmunity and Generates Regulatory T Cell Precursors. Nat Immunol (2016) 17(3):304–14. 10.1038/ni.3331 PMC4755884 26829766

[44] Barton BM Xu R Wherry EJ Porrett PM. Pregnancy Promotes Tolerance to Future Offspring by Programming Selective Dysfunction in Long-Lived Maternal T Cells. J Leukoc Biol (2017) 101(4):975–87. 10.1189/jlb.1A0316-135R PMC12040000 27810945

[45] Kinder JM Turner LH Stelzer IA Miller-Handley H Burg A Shao TY. Cd8(+) T Cell Functional Exhaustion Overrides Pregnancy-Induced Fetal Antigen Alloimmunization. Cell Rep (2020) 31(12):107784. 10.1016/j.celrep.2020.107784 32579916 PMC7383938

[46] Suah AN Tran DV Khiew SH Andrade MS Pollard JM Jain D. Pregnancy-Induced Humoral Sensitization Overrides T Cell Tolerance to Fetus-Matched Allografts in Mice. J Clin Invest (2021) 131(1):e140715. 10.1172/JCI140715 PMC7773355 33393512

[47] Kinder JM Stelzer IA Arck PC Way SS. Immunological Implications of Pregnancy-Induced Microchimerism. Nat Rev Immunol (2017) 17(8):483–94. 10.1038/nri.2017.38 PMC5532073 28480895

[48] Higgins R Lowe D Daga S Hathaway M Williams C Lam FT. Pregnancy-Induced HLA Antibodies Respond More Vigorously After Renal Transplantation Than Antibodies Induced by Prior Transplantation. Hum Immunol (2015) 76(8):546–52. 10.1016/j.humimm.2015.06.013 26116896

[49] Collins MK Tay CS Erlebacher A. Dendritic Cell Entrapment Within the Pregnant Uterus Inhibits Immune Surveillance of the Maternal/Fetal Interface in Mice. J Clin Invest (2009) 119(7):2062–73. 10.1172/JCI38714 PMC2701881 19546507

[50] Heesters BA van der Poel CE Das A Carroll MC. Antigen Presentation to B Cells. Trends Immunol (2016) 37(12):844–54. 10.1016/j.it.2016.10.003 27793570

[51] Pipi E Nayar S Gardner DH Colafrancesco S Smith C Barone F. Tertiary Lymphoid Structures: Autoimmunity Goes Local. Front Immunol (2018) 9:1952. 10.3389/fimmu.2018.01952 30258435 PMC6143705

[52] Lund FE Partida-Sanchez S Lee BO Kusser KL Hartson L Hogan RJ. Lymphotoxin-Alpha-Deficient Mice Make Delayed, But Effective, T and B Cell Responses to Influenza. J Immunol (2002) 169(9):5236–43. 10.4049/jimmunol.169.9.5236 12391242

[53] Cyster JG. B Cell Follicles and Antigen Encounters of the Third Kind. Nat Immunol (2010) 11(11):989–96. 10.1038/ni.1946 20959804

[54] Phan TG Grigorova I Okada T Cyster JG. Subcapsular Encounter and Complement-Dependent Transport of Immune Complexes by Lymph Node B Cells. Nat Immunol (2007) 8(9):992–1000. 10.1038/ni1494 17660822

[55] Heesters BA Carroll MC. The Role of Dendritic Cells in s. Pneumoniae Transport to Follicular Dendritic Cells. Cell Rep (2016) 16(12):3130–7. 10.1016/j.celrep.2016.08.049 PMC5790206 27653679

[56] Heesters BA Chatterjee P Kim YA Gonzalez SF Kuligowski MP Kirchhausen T. Endocytosis and Recycling of Immune Complexes by Follicular Dendritic Cells Enhances B Cell Antigen Binding and Activation. Immunity (2013) 38(6):1164–75. 10.1016/j.immuni.2013.02.023 PMC3773956 23770227

[57] Gatto D Brink R. B Cell Localization: Regulation by EBI2 and its Oxysterol Ligand. Trends Immunol (2013) 34(7):336–41. 10.1016/j.it.2013.01.007 23481574

[58] Reif K Ekland EH Ohl L Nakano H Lipp M Forster R. Balanced Responsiveness to Chemoattractants From Adjacent Zones Determines B-Cell Position. Nature (2002) 416(6876):94–9. 10.1038/416094a 11882900

[59] Zhang Y Garcia-Ibanez L Toellner KM. Regulation of Germinal Center B-Cell Differentiation. Immunol Rev (2016) 270(1):8–19. 10.1111/imr.12396 26864101 PMC4755139

[60] Obukhanych TV Nussenzweig MC. T-Independent Type Ii Immune Responses Generate Memory B Cells. J Exp Med (2006) 203(2):305–10. 10.1084/jem.20052036 PMC2118207 16476769

[61] Parker DC. T Cell-Dependent B Cell Activation. Annu Rev Immunol (1993) 11:331–60. 10.1146/annurev.iy.11.040193.001555 8476565

[62] Goenka R Barnett LG Silver JS O’Neill PJ Hunter CA Cancro MP. Cutting Edge: Dendritic Cell-Restricted Antigen Presentation Initiates the Follicular Helper T Cell Program But Cannot Complete Ultimate Effector Differentiation. J Immunol (2011) 187(3):1091–5. 10.4049/jimmunol.1100853 PMC3171798 21715693

[63] Yu D Rao S Tsai LM Lee SK He Y Sutcliffe EL. The Transcriptional Repressor Bcl-6 Directs T Follicular Helper Cell Lineage Commitment. Immunity (2009) 31(3):457–68. 10.1016/j.immuni.2009.07.002 19631565

[64] Nurieva RI Chung Y Hwang D Yang XO Kang HS Ma L. Generation of T Follicular Helper Cells is Mediated by Interleukin-21 But Independent of T Helper 1, 2, or 17 Cell Lineages. Immunity (2008) 29(1):138–49. 10.1016/j.immuni.2008.05.009 PMC2556461 18599325

[65] Elgueta R Benson MJ de Vries VC Wasiuk A Guo Y Noelle RJ. Molecular Mechanism and Function of CD40/CD40L Engagement in the Immune System. Immunol Rev (2009) 229(1):152–72. 10.1111/j.1600-065X.2009.00782.x PMC3826168 19426221

[66] Lee SK Rigby RJ Zotos D Tsai LM Kawamoto S Marshall JL. B Cell Priming for Extrafollicular Antibody Responses Requires Bcl-6 Expression by T Cells. J Exp Med (2011) 208(7):1377–88. 10.1084/jem.20102065 PMC3135363 21708925

[67] Crotty S. Follicular Helper Cd4 T Cells (Tfh). Annu Rev Immunol (2011) 29:621–63. 10.1146/annurev-immunol-031210-101400 21314428

[68] Ma CS Phan TG. Here, There and Everywhere: T Follicular Helper Cells on the Move. Immunology (2017) 152(3):382–7. 10.1111/imm.12793 PMC5629422 28704588

[69] Coffey F Alabyev B Manser T. Initial Clonal Expansion of Germinal Center B Cells Takes Place at the Perimeter of Follicles. Immunity (2009) 30(4):599–609. 10.1016/j.immuni.2009.01.011 19303334 PMC2716660

[70] Akkaya M Kwak K Pierce SK. B Cell Memory: Building Two Walls of Protection Against Pathogens. Nat Rev Immunol (2020) 20(4):229–38. 10.1038/s41577-019-0244-2 PMC7223087 31836872

[71] Paus D Phan TG Chan TD Gardam S Basten A Brink R. Antigen Recognition Strength Regulates the Choice Between Extrafollicular Plasma Cell and Germinal Center B Cell Differentiation. J Exp Med (2006) 203(4):1081–91. 10.1084/jem.20060087 PMC2118299 16606676

[72] Schwickert TA Victora GD Fooksman DR Kamphorst AO Mugnier MR Gitlin AD. A Dynamic T Cell-Limited Checkpoint Regulates Affinity-Dependent B Cell Entry Into the Germinal Center. J Exp Med (2011) 208(6):1243–52. 10.1084/jem.20102477 PMC3173244 21576382

[73] Kwak K Quizon N Sohn H Saniee A Manzella-Lapeira J Holla P. Intrinsic Properties of Human Germinal Center B Cells Set Antigen Affinity Thresholds. Sci Immunol (2018) 3(29):eaau6598. 10.1126/sciimmunol.aau6598 30504208 PMC6314460

[74] Akkaya M Akkaya B Kim AS Miozzo P Sohn H Pena M. Toll-Like Receptor 9 Antagonizes Antibody Affinity Maturation. Nat Immunol (2018) 19(3):255–66. 10.1038/s41590-018-0052-z PMC5839995 29476183

[75] Gatto D Wood K Brink R. EBI2 Operates Independently of But in Cooperation With CXCR5 and CCR7 to Direct B Cell Migration and Organization in Follicles and the Germinal Center. J Immunol (2011) 187(9):4621–8. 10.4049/jimmunol.1101542 21948984

[76] Park CS Choi YS. How do Follicular Dendritic Cells Interact Intimately With B Cells in the Germinal Centre? Immunology (2005) 114(1):2–10. 10.1111/j.1365-2567.2004.02075.x 15606789 PMC1782056

[77] Victora GD Schwickert TA Fooksman DR Kamphorst AO Meyer-Hermann M Dustin ML. Germinal Center Dynamics Revealed by Multiphoton Microscopy With a Photoactivatable Fluorescent Reporter. Cell (2010) 143(4):592–605. 10.1016/j.cell.2010.10.032 21074050 PMC3035939

[78] Liu D Xu H Shih C Wan Z Ma X Ma W. T-B-Cell Entanglement and ICOSL-Driven Feed-Forward Regulation of Germinal Centre Reaction. Nature (2015) 517(7533):214–8. 10.1038/nature13803 25317561

[79] Goenka R Matthews AH Zhang B O’Neill PJ Scholz JL Migone TS. Local Blys Production by T Follicular Cells Mediates Retention of High Affinity B Cells During Affinity Maturation. J Exp Med (2014) 211(1):45–56. 10.1084/jem.20130505 24367004 PMC3892970

[80] Seki M Gearhart PJ Wood RD. DNA Polymerases and Somatic Hypermutation of Immunoglobulin Genes. EMBO Rep (2005) 6(12):1143–8. 10.1038/sj.embor.7400582 PMC1369213 16319960

[81] Roco JA Mesin L Binder SC Nefzger C Gonzalez-Figueroa P Canete PF. Class-Switch Recombination Occurs Infrequently in Germinal Centers. Immunity (2019) 51(2):337–50 e7. 10.1016/j.immuni.2019.07.001 31375460 PMC6914312

[82] Shinnakasu R Inoue T Kometani K Moriyama S Adachi Y Nakayama M. Regulated Selection of Germinal-Center Cells Into the Memory B Cell Compartment. Nat Immunol (2016) 17(7):861–9. 10.1038/ni.3460 27158841

[83] Weisel FJ Zuccarino-Catania GV Chikina M Shlomchik MJ. A Temporal Switch in the Germinal Center Determines Differential Output of Memory B and Plasma Cells. Immunity (2016) 44(1):116–30. 10.1016/j.immuni.2015.12.004 PMC4724390 26795247

[84] Purtha WE Tedder TF Johnson S Bhattacharya D Diamond MS. Memory B Cells, But Not Long-Lived Plasma Cells, Possess Antigen Specificities for Viral Escape Mutants. J Exp Med (2011) 208(13):2599–606. 10.1084/jem.20110740 PMC3244041 22162833

[85] Andrade P Gimblet-Ochieng C Modirian F Collins M Cardenas M Katzelnick LC. Impact of Pre-Existing Dengue Immunity on Human Antibody and Memory B Cell Responses to Zika. Nat Commun (2019) 10(1):938. 10.1038/s41467-019-08845-3 30808875 PMC6391383

[86] Elsner RA Shlomchik MJ. Germinal Center and Extrafollicular B Cell Responses in Vaccination, Immunity, and Autoimmunity. Immunity (2020) 53(6):1136–50. 10.1016/j.immuni.2020.11.006 PMC7748291 33326765

[87] Cunningham AF Gaspal F Serre K Mohr E Henderson IR Scott-Tucker A. Salmonella Induces a Switched Antibody Response Without Germinal Centers That Impedes the Extracellular Spread of Infection. J Immunol (2007) 178(10):6200–7. 10.4049/jimmunol.178.10.6200 17475847

[88] Popescu M Cabrera-Martinez B Winslow GM. TNF-Alpha Contributes to Lymphoid Tissue Disorganization and Germinal Center B Cell Suppression During Intracellular Bacterial Infection. J Immunol (2019) 203(9):2415–24. 10.4049/jimmunol.1900484 PMC6810925 31570507

[89] Jenks SA Cashman KS Zumaquero E Marigorta UM Patel AV Wang X. Distinct Effector B Cells Induced by Unregulated Toll-Like Receptor 7 Contribute to Pathogenic Responses in Systemic Lupus Erythematosus. Immunity (2018) 49(4):725–39 e6. 10.1016/j.immuni.2018.08.015 30314758 PMC6217820

[90] Toyama H Okada S Hatano M Takahashi Y Takeda N Ichii H. Memory B Cells Without Somatic Hypermutation are Generated From Bcl6-Deficient B Cells. Immunity (2002) 17(3):329–39. 10.1016/s1074-7613(02)00387-4 12354385

[91] Takahashi Y Dutta PR Cerasoli DM Kelsoe G. *In Situ* Studies of the Primary Immune Response to (4-Hydroxy-3-Nitrophenyl)Acetyl. V. Affinity Maturation Develops in Two Stages of Clonal Selection. J Exp Med (1998) 187(6):885–95. 10.1084/jem.187.6.885 PMC2212188 9500791

[92] Bohannon C Powers R Satyabhama L Cui A Tipton C Michaeli M. Long-Lived Antigen-Induced IgM Plasma Cells Demonstrate Somatic Mutations and Contribute to Long-Term Protection. Nat Commun (2016) 7:11826. 10.1038/ncomms11826 27270306 PMC4899631

[93] Cattoretti G Buttner M Shaknovich R Kremmer E Alobeid B Niedobitek G. Nuclear and Cytoplasmic AID in Extrafollicular and Germinal Center B Cells. Blood (2006) 107(10):3967–75. 10.1182/blood-2005-10-4170 16439679

[94] Trivedi N Weisel F Smita S Joachim S Kader M Radhakrishnan A. Liver is a Generative Site for the B Cell Response to Ehrlichia Muris. Immunity (2019) 51(6):1088–101.e5. 10.1016/j.immuni.2019.10.004 31732168 PMC6955021

[95] Lavinder JJ Wine Y Giesecke C Ippolito GC Horton AP Lungu OI. Identification and Characterization of the Constituent Human Serum Antibodies Elicited by Vaccination. Proc Natl Acad Sci U S A (2014) 111(6):2259–64. 10.1073/pnas.1317793111 PMC3926051 24469811

[96] Wrammert J Koutsonanos D Li GM Edupuganti S Sui J Morrissey M. Broadly Cross-Reactive Antibodies Dominate the Human B Cell Response Against 2009 Pandemic H1N1 Influenza Virus Infection. J Exp Med (2011) 208(1):181–93. 10.1084/jem.20101352 PMC3023136 21220454

[97] Zuccarino-Catania GV Sadanand S Weisel FJ Tomayko MM Meng H Kleinstein SH. CD80 and PD-L2 Define Functionally Distinct Memory B Cell Subsets That are Independent of Antibody Isotype. Nat Immunol (2014) 15(7):631–7. 10.1038/ni.2914 PMC4105703 24880458

[98] Mesin L Schiepers A Ersching J Barbulescu A Cavazzoni CB Angelini A. Restricted Clonality and Limited Germinal Center Reentry Characterize Memory B Cell Reactivation by Boosting. Cell (2020) 180(1):92–106 e11. 10.1016/j.cell.2019.11.032 31866068 PMC6958527

[99] Turner JS Zhou JQ Han J Schmitz AJ Rizk AA Alsoussi WB. Human Germinal Centres Engage Memory and Naive B Cells After Influenza Vaccination. Nature (2020) 586(7827):127–32. 10.1038/s41586-020-2711-0 PMC7566073 32866963

[100] Andrews SF Huang Y Kaur K Popova LI Ho IY Pauli NT. Immune History Profoundly Affects Broadly Protective B Cell Responses to Influenza. Sci Transl Med (2015) 7(316):316ra192. 10.1126/scitranslmed.aad0522 PMC4770855 26631631

[101] Seifert M Przekopowitz M Taudien S Lollies A Ronge V Drees B. Functional Capacities of Human IgM Memory B Cells in Early Inflammatory Responses and Secondary Germinal Center Reactions. Proc Natl Acad Sci USA (2015) 112(6):E546–55. 10.1073/pnas.1416276112 PMC4330750 25624468

[102] Pape KA Taylor JJ Maul RW Gearhart PJ Jenkins MK. Different B Cell Populations Mediate Early and Late Memory During an Endogenous Immune Response. Science (2011) 331(6021):1203–7. 10.1126/science.1201730 PMC3993090 21310965

[103] Karahan GE de Vaal YJ Roelen DL Buchli R Claas FH Heidt S. Quantification of HLA Class II-Specific Memory B Cells in HLA-Sensitized Individuals. Hum Immunol (2015) 76(2-3):129–36. 10.1016/j.humimm.2015.01.014 25636565

[104] Heidt S Roelen DL de Vaal YJ Kester MG Eijsink C Thomas S. A Novel ELISPOT Assay to Quantify HLA-Specific B Cells in HLA-Immunized Individuals. Am J Transplant (2012) 12(6):1469–78. 10.1111/j.1600-6143.2011.03982.x 22390272

[105] Karahan GE de Vaal YJH Krop J Wehmeier C Roelen DL Claas FHJ. A Memory B Cell Crossmatch Assay for Quantification of Donor-Specific Memory B Cells in the Peripheral Blood of HLA-Immunized Individuals. Am J Transplant (2017) 17(10):2617–26. 10.1111/ajt.14293 28371365

[106] Lucia M Luque S Crespo E Melilli E Cruzado JM Martorell J. Preformed Circulating HLA-Specific Memory B Cells Predict High Risk of Humoral Rejection in Kidney Transplantation. Kidney Int (2015) 88(4):874–87. 10.1038/ki.2015.205 26176829

[107] Snanoudj R Claas FH Heidt S Legendre C Chatenoud L Candon S. Restricted Specificity of Peripheral Alloreactive Memory B Cells in HLA-Sensitized Patients Awaiting a Kidney Transplant. Kidney Int (2015) 87(6):1230–40. 10.1038/ki.2014.390 25565312

[108] Karahan GE Krop J Wehmeier C de Vaal YJH Langerak-Langerak J Roelen DL. An Easy and Sensitive Method to Profile the Antibody Specificities of HLA-Specific Memory B Cells. Transplantation (2019) 103(4):716–23. 10.1097/TP.0000000000002516 PMC6430593 30418423

[109] Zachary AA Kopchaliiska D Montgomery RA Leffell MS. HLA-Specific B Cells: I. A Method for Their Detection, Quantification, and Isolation Using HLA Tetramers. Transplantation (2007) 83(7):982–8. 10.1097/01.tp.0000259017.32857.99 17460571

[110] Zachary AA Kopchaliiska D Montgomery RA Melancon JK Leffell MS. HLA-Specific B Cells: II. Application to Transplantation. Transplantation (2007) 83(7):989–94. 10.1097/01.tp.0000259019.68244.d7 17460572

[111] Mulder A Eijsink C Kardol MJ Franke-van Dijk ME van der Burg SH Kester M. Identification, Isolation, and Culture of HLA-A2-Specific B Lymphocytes Using MHC Class I Tetramers. J Immunol (2003) 171(12):6599–603. 10.4049/jimmunol.171.12.6599 14662862

[112] Luque S Lucia M Melilli E Lefaucheur C Crespo M Loupy A. Value of Monitoring Circulating Donor-Reactive Memory B Cells to Characterize Antibody-Mediated Rejection After Kidney Transplantation. Am J Transplant (2019) 19(2):368–80. 10.1111/ajt.15055 30085394

[113] Goel RR Apostolidis SA Painter MM Mathew D Pattekar A Kuthuru O. Distinct Antibody and Memory B Cell Responses in SARS-Cov-2 Naive and Recovered Individuals Following Mrna Vaccination. Sci Immunol (2021) 6(58):eabi6950. 10.1126/sciimmunol.abi6950 33858945 PMC8158969

[114] Gaebler C Wang Z Lorenzi JCC Muecksch F Finkin S Tokuyama M. Evolution of Antibody Immunity to SARS-Cov-2. Nature (2021) 591(7851):639–44. 10.1038/s41586-021-03207-w PMC8221082 33461210

[115] Khiew SH Jain D Chen J Yang J Yin D Young JS. Transplantation Tolerance Modifies Donor-Specific B Cell Fate to Suppress De Novo Alloreactive B Cells. J Clin Invest (2020) 130(7):3453–66. 10.1172/JCI132814 PMC7329196 32452834

[116] Lau D Lan LY Andrews SF Henry C Rojas KT Neu KE. Low CD21 Expression Defines a Population of Recent Germinal Center Graduates Primed for Plasma Cell Differentiation. Sci Immunol (2017) 2(7):eaai8153. 10.1126/sciimmunol.aai8153 28783670 PMC5896567

[117] Nellore A Zumaquero E Scharer CD King RG Tipton CM Fucile CF. Influenza-Specific Effector Memory B Cells Predict Long-Lived Antibody Responses to Vaccination in Humans. bioRxiv (2021) 643973. 10.1101/643973 PMC10113805 36958335

